# Age-Related Choroidal Involution Is Associated with the Senescence of Endothelial Progenitor Cells in the Choroid

**DOI:** 10.3390/biomedicines12122669

**Published:** 2024-11-22

**Authors:** Ali Riza Nazari, Loraine Gresseau, Tiffany Habelrih, Aliabbas Zia, Isabelle Lahaie, Yosra Er-Reguyeg, France Coté, Borhane Annabi, Alain Rivard, Sylvain Chemtob, Michel Desjarlais

**Affiliations:** 1 Department of Ophthalmology, Maisonneuve-Rosemont Hospital Research Center, University of Montréal, Montréal, QC H1T 2M4, Canada; 2 Department of Pediatrics, Ophthalmology and Pharmacology, Centre Hospitalier Universitaire Sainte-Justine Research Center, Montréal, QC H2X 0A9, Canada; 3 Département de Chimie, Université du Québec à Montréal, Montréal, QC H3C 3P8, Canada; 4 Department of Medicine, Centre Hospitalier de l’Université de Montréal (CHUM) Research Center, Montréal, QC H2X 0A9, Canada

**Keywords:** aging, choroidal involution, endothelial progenitor cell (EPC), vascular network, senescence

## Abstract

**Background:** Choroidal involution is a common feature of age-related ischemic retinopathies such as age-related macular degeneration (AMD). It is now well recognized that endothelial progenitor cells (EPCs) are essential to endothelial repair processes and in maintaining vascular integrity. However, the contribution of EPCs and the role of senescence in age-related choroidal vascular degeneration remain to be investigated. In this study, we compared the senescent phenotype of EPCs in the choroid and performed whole-genome profiling of EPCs derived from young versus old rats. **Methods and Results:** We isolated and compared the retinas of young (6-weeks-old) and old (16–18-month-old) rats. The thickness of the choroid and outer nuclear layer (ONL), along with local quantification of CD34+ EPCs, was performed. Compared to young rats, older rats displayed a significant reduction in choroidal and ONL thickness associated with markedly fewer choroid-localized EPCs; this was attested by lower expression of several EPC markers (CXCR4, CD34, CD117, CD133, and KLF-2). Choroid and choroid-localized EPCs displayed abundant senescence as revealed by increased β-gal and P53 expression and decreased Lamin-B1 (immunostaining and RT-qPCR). Concordantly, choroidal cells and EPCs isolated from older rats were unable to form vascular networks ex vivo. To better understand the potential mechanisms associated with the dysfunctional EPCs linked to age-related choroidal involution, we performed whole-genome profiling (mRNA and miRNA) of EPCs derived from old and young rats using next-generation sequencing (NGS); 802 genes were significantly modulated in old vs. young EPCs, corresponding to ~2% of total genes expressed. Using a bioinformatic algorithm, the KEGG pathways suggested that these genes participate in the modulation of several key signaling processes including inflammation, G protein-coupled receptors, and hematopoietic cell lineages. Moreover, we identified 13 miRNAs involved in the regulation of immune system processes, cell cycle arrest and senescence, which are significantly modulated in EPCs from old rats compared to young ones. **Conclusions:** Our results suggest that age-related choroidal involution is associated with fewer EPCs, albeit displaying a senescence-like phenotype. One would be tempted to propose that biological modification of native EPCs (such as with senolytic agents) could potentially provide a new strategy to preserve the vascular integrity of the aged choroid, and evade progression to degenerative maculopathies.

## 1. Introduction

Choroidal involution is a key component of the pathogenesis of ischemic retinopathies that can lead to blindness including retinopathy of prematurity (ROP) and age-related macular degeneration (AMD). Involution of the choroid is also observed with aging, and as such possibly contributes to age-associated degenerative processes [1]. Over the past several years, studies have largely focused on the pathological neovascularization of ROP and AMD, while few groups have looked at choroidal involution, particularly in the context of aging. In healthy adults, the choroidal vasculature is an expansive vascular network responsible for 90% of the retinal distribution of blood flow from the ophthalmic artery. Choroidal blood flow is considered the highest in the body [2] and accordingly has long been assumed to be protected from choroidal ischemia. However, it has recently been reported that several conditions, such as ROP and AMD, involve an important loss of the choroidal vasculature, resulting in significant choroidal ischemia [3,4]. Consequently, choroidal ischemia impairs the function of distinct cells, including endothelial cells (ECs), the retinal pigment epithelium (RPE), and photoreceptors [5], all of which are in the outer and sub-retina. The preservation of vascular integrity depends not only on healthy endothelial cells, but also involves the active contribution of endothelial progenitor cells (EPCs); EPCs play a prominent role in development and in repair processes, maintaining the well-being of vascular networks [6,7,8]. 

In ischemic tissues, EPCs have the ability to directly integrate the endothelium to form their own vessels (the process of vasculogenesis), while they can also secrete proangiogenic factors, such as VEGF, EPO, SDF-1, and PDGF, to elicit paracrine actions on indwelling ECs [9]. EPCs have been shown to promote neovascularization and post-ischemic revascularization in animal models of tissue ischemia [9,10,11]. Intriguingly, similar phenomena linking EPC failure and choroidal involution have been reported both in the context of prematurity and during aging. Based on previous studies demonstrating that elevated oxidative stress levels and sustained inflammation actively contribute to the choroidal degeneration and dysfunction of EPCs, our group recently showed that oxidative stress leads to the reduced number and dysfunction of EPCs in a rat model of ROP (oxygen-induced retinopathy) [8,11]; this phenotype is also found in choroidal involution linked to aging [12,13]. A reduction in circulating EPCs in aging patients as well as in subjects presenting inflammatory conditions has also been reported [14]; moreover EPCs extracted from aging animals possess limited vasculogenic activity. 

However, the specific role of EPCs in choroidal involution during aging and the potential mechanisms that are involved remain to be determined. Considering that senescence is an important physiological process found during cellular aging, we postulated that the development of senescence in EPCs localized in the aged choroid could compromise its angiogenic activity and participate in the inability of the choroid to repair once subjected to involution. In this context, replicative senescence, which compromises cell cycle, obligatorily limits cell proliferation and migration [15]. Senescent cells are also characterized by important metabolic changes through the adoption of a pro-inflammatory phenotype [16]. These changes contribute to age-related disorders that apply not only to vascular degeneration, but also inflammation and cancer [17]. We hypothesize that EPCs present in the choroid exhibit a senescent phenotype during aging, likely contributing to the curtailed repair of this tissue during age-related degeneration. For the first time, we hereby quantify numbers of senescent CD34+/CD133+ EPCs localized in the choroid of older rats relative to younger subjects and perform whole-genome profiling (mRNA and miRNA) of EPCs derived from old and young rats using next-generation sequencing (NGS) to elucidate potential mechanisms associated with the phenotype. 

## 2. Materials and Methods

### 2.1. Animal Care

All animal experimental procedures were performed with strict adherence to the ARVO Statement for the Use of Animals in Ophthalmic and Vision Research and approved by the Animal Care Committee of the Hospital Maisonneuve-Rosemont (2022–2878) in accordance with guidelines established by the Canadian Council on Animal Care. 

### 2.2. Rat Model of Age-Related Choroidal Involution and Isolation of EPCs

Age-related choroidal involution was studied using young Sprague Dawley rats (3–4 months old) and compared to older rats (18–20 months old), consistent with what is observed in aging humans, and placing these at risk of outer retinal degeneration. Three to five animals (*n* = 3 for molecular analysis; qRT-PCR and NGS and *n* = 5 for immunostaining analysis) per group (young and old) were used and their eyes were collected for immunostaining (left eye) and molecular analysis (right eye). Bone marrow-derived endothelial progenitor cells EPCs (BM-EPCs) were isolated from the same two groups of rats as previously described, [8] to perform NGS analysis as well as vasculogenic and senescence assays. Briefly, to obtain primary EPCs culture, the mononuclear cells were isolated from the femoral and tibial bone marrow. The bone marrow was cut in the bone extremity and flushed using a 5CC needle syringe with medium 200 (Thermos Fisher Scientific, Mississauga, ON, Canada) supplemented with 10% fetal bovine serum (FBS, Wisent, St-Jean-Baptiste, QC, Canada), 100 IU/mL penicillin, 0.1 mg/mL streptomycin (Wisent, St-Jean-Baptiste, QC, Canada), and low serum growth supplement (LSGS; 2% FBS, 3 ng/mL bFGF, 10 mg/mL heparin, 1 mg/mL hydrocortisone, and 10 ng/mL EGF; Thermos Fisher Scientific, Mississauga, ON, Canada). The cell suspension was then centrifuged at 1500 RPM for 5 min and the cell pellet was resuspended in red blood cell lysis buffer (1× diluted in PBS). After a second centrifugation, the red blood cell-free pellet was resuspended in EPC culture medium and plated in a density of 5 million cells in a T25 fibronectin-precoated plates (MilliporeSigma, Burlington, MA, USA) and kept for 21 days in culture. The BM-EPCs, also known as «late outgrowth EPCs» or «endothelial colony forming cells» (ECFCs) express hematopoietic stem cell myeloid markers, such as CD34 and CD117, and endothelial markers, such as lectin, VEGFR2, and CD31. The cells showing EC phenotype positivity for lectin and forming tubes were considered as EPCs [8].

### 2.3. Immunohistochemistry of Choroidal Vessels

In order to study the choroidal vasculature, eyes were fixed overnight in 4% paraformaldehyde (MilliporeSigma, Burlington, MA, USA) and then rinsed twice with PBS. The cornea and lens were gently removed from the eye. Posterior eyecups were kept in 30% sucrose overnight and embedded in optimal cutting temperature (OCT) medium (Leica Biosystems, Concord, ON, Canada). The frozen sections of the posterior eyecups along the optic nerve were cut into 10 μm sagittal sections. Choroidal cryosections were washed with PBS solution containing 0.1% Triton X-100 for 30 min and then incubated overnight in the dark to stain the choroidal vessels with a PBS solution consisting of 1 mM CaCl_2_, 0.1% Triton X-100 and 1:100 of Griffonia (Bandeiraea) Simplicifolia Lectin I (Vectors Lab, Newark, CA, USA). In some experiments, retinal cryosections were co-stained with (lectin/CD34, lectin/CD133, lectin/p53 or CD34/p53) by adding a recombinant Anti-CD34 antibody 1:200 (Abcam, Cambridge, UK; Royaume-Uni, (ab81289)), a polyclonal rabbit anti-CD133 (Thermos Fisher Scientific, Mississauga, ON, Canada (PA5-38014)), or a p53 antibody 1:100 (Santa Cruz Biotechnology, Dallas, TX, USA, (SC-1313), and incubated overnight at 4 °C in the blocking solution. Secondary antibodies, such as Alexa Fluor 594 anti-rabbit or Alexa Fluor 647 anti-goat (Thermos Fisher Scientific, Mississauga, ON, Canada), were used at a dilution of 1:500 to detect CD34 or P53, respectively. Cell nuclei were identified with 4,6-diamidino-2-phenylindole (DAPI) labeling (MilliporeSigma, Burlington, MA, USA). An incubation using rabbit or goat IgG as a primary antibody was conducted as a negative control. Cryosections were then visualized with an epifluorescence microscope (Eclipse E800; Nikon, Tokyo, Japan). The image was split into the three-color channels (RGB Merge/split function) to obtain one image per channel using Image J. At defined distances from the optic nerve (starting at the 0 mm position), ten measurements per retinal and choroidal sections were performed on at least two centrals cryosections (with optic nerve) per eye, as demonstrated in Supplementary Figure S6 [18]. The analysis of layer thickness was performed using ImageJ software (http://imagej.nih.gov/, accessed on 1 January 2023). The area under the curve was integrated using the statistical analysis program (GraphPad Prism software version 5.01).

### 2.4. Senescence Analysis Using β-Gal Assay

SA-β-gal activity was detected with the Senescence beta-Galactosidase Staining Kit (Cell Signaling Technology, Danvers, MA, USA) according to the manufacturer’s protocol. Briefly, isolated EPCs were washed twice with PBS before being fixed in 4% paraformaldehyde fixative solution for 15 min at room temperature. EPCs or cryosections were then washed twice with PBS 1X and stained in β-galactosidase Staining Solution overnight at 37 °C. β-Gal-positive cells developed a blue-green colored precipitate.

### 2.5. EPC Capillary-Like Tubulogenesis and Choroidal Angiogenic Sprouting Assay on Matrigel

The capacity of EPCs to form capillary-like tubules was evaluated using a Matrigel assay. EPCs were plated at a density of 30,000 cells/well in 96-well plates precoated with a growth factor-reduced Matrigel Matrix (Corning Inc., Corning, NY, USA) and cultured at 37 °C for 6 h in normoxia in complete endothelial growth medium containing LSGS (this medium contains an angiogenic growth factor including: 3 ng/mL bFGF, 5 ng/mL IGF-1, and 10 ng/mL EGF; Thermos Fisher Scientific, Mississauga, ON, Canada). A light microscope at a magnification of 10× was used to take pictures of the capillary-like tubes; these were quantified by counting branches. The angiogenic sprouting capacity of the isolated rat choroid was assessed as previously described [19]. Briefly, the choroid was sectioned into 1 mm pieces and placed in growth factor-reduced Matrigel (Corning, NY, USA) in 96-well plates and cultured in 37 °C, 5% CO_2_ for 5 days in endothelial Cell Growth Medium-2 (EGM-2) (Lonza Bioscience, Walkersville, MD, USA). Photomicrographs of individual explants were taken at day 5 using an inverted phase-contrast microscope (AxioObserver; Zeiss, Toronto, ON, Canada) and microvascular sprouting (the total area occupied by vessel sprouts, excluding the explant) was quantified using Image J.

### 2.6. Reverse Transcription–Quantitative Polymerase Chain Reaction (RT-qPCR) Analyses

To compare the choroidal mRNA expression levels of EPCs and senescent markers between the two groups of rats, the RNeasy mini kit (Qiagen, Toronto, ON, Canada) was used to extract the total RNA of the whole choroid according to the manufacturer’s protocol. To generate cDNA, the iScript-II RT kit (Biorad, Mississauga, ON, Canada) was used for reverse transcription as per the manufacturer’s protocol. In total, 25 ng of cDNA sample, 2 µM of specific primers (Integrated DNA Technologies, Coralville, IA, USA) for the selected mRNAs, and Universal SYBR Green Supermix (Biorad, Mississauga, ON, Canada) were used for reverse transcription–quantitative polymerase chain reaction. The instrument detection system ABI Prism 7500 (Applied Biosystems, Foster City, CA, USA) calculated the relative expression that was normalized to beta-Actin and GAPDH. The list of primer sequences is available in Supplementary Table S1. 

### 2.7. Next-Generation Sequencing (NGS) and Predictive Pathway Analysis

EPCs were isolated from the different groups of rats (*n* = 3 rats per groups; young or old) and cultured for 21 days (see EPCs isolation in previous section). Briefly, for mRNAseq, total RNA was quantified using Qubit (Thermo Scientific) and the quality was assessed with the 2100 Bioanalyzer (Agilent Technologies, Mississauga, ON, Canada). Transcriptome libraries were generated using the KAPA RNA HyperPrep (Roche, Laval, QC, Canada) using a poly-A selection (Thermo Scientific). Sequencing was performed on the Illumina NextSeq500, obtaining around 25 M reads per sample. For miRNAseq, small RNA was quantified using Qubit (Thermo Scientific) and the quality was assessed with the 2100 Bioanalyzer (Agilent Technologies). The libraries were prepared using the QIAseq miRNA Library Kit (Qiagen). Sequencing was performed on an Illumina NextSeq 500, obtaining around 5 million reads per sample. The global analysis to identify the major pathways altered by the top significant modulated genes (mRNA) in old EPCs was conducted using Enrichr software (https://maayanlab.cloud/Enrichr/, accessed on 1 January 2023) [20]. The predicted pathway affected by the selected miRNA was performed as previously described [14] using the Kyoto Encyclopedia of Genes and Genomes (KEGG) and the dendrogram clusters of the predictive biological Gene Ontology (GO) process-connectome of selected miRs were analyzed using the DIANA tool program (http://diana.imis.athena-innovation.gr/DianaTools/index.php, accessed on 1 January 2023) [21]. In addition, to establish a global expression profile in old EPCs, this NGS study aimed to focus the analyses particularly on the inflammatory genes, including the interleukin family (IL-1, IL-6, TNF-α), reported to be increased in aging and EPC dysfunction.

### 2.8. Statistical Analysis

All results are presented as mean ± SEM. Statistical significance was evaluated by a one- or two-way ANOVA followed by a Bonferroni post hoc test. A value of *p* < 0.05 was interpreted to denote statistical significance.

## 3. Results

Age-related choroidal vascular involution is associated with decreased numbers of EPCs in the choroid and acquisition of a senescence phenotype.

**Figure 1:**
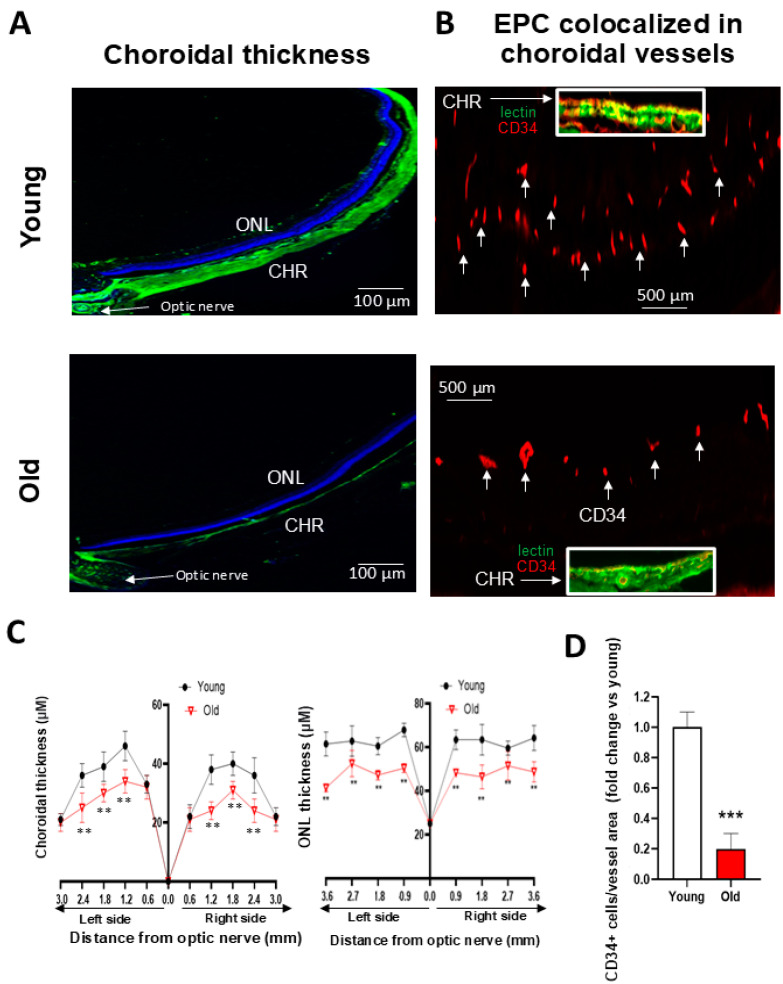
Choroidal thickness and EPC number in young compared to old choroids. (**A**,**C**) Representative images (**A**) and quantitative analyses (**C**) of choroidal (CHR) and photoreceptor (OPL) thickness in retinal cross-sections stained for vessels (isolectin-positive, green) and DAPI in old vs. young rats. (**B**,**D**) EPCs (CD34+ cells) co-localized in choroidal vessels in old vs. young rats. For the choroid of old animals in the inset in (**B**), the magnification is 10-fold greater than that in young animals to make it possible to compare CD34 expression over the same choroidal vessel area. Data are mean ± SEM. ** *p* < 0.01 or *** *p* < 0.001 vs. young rats. *n* = 5.

**Figure 2:**
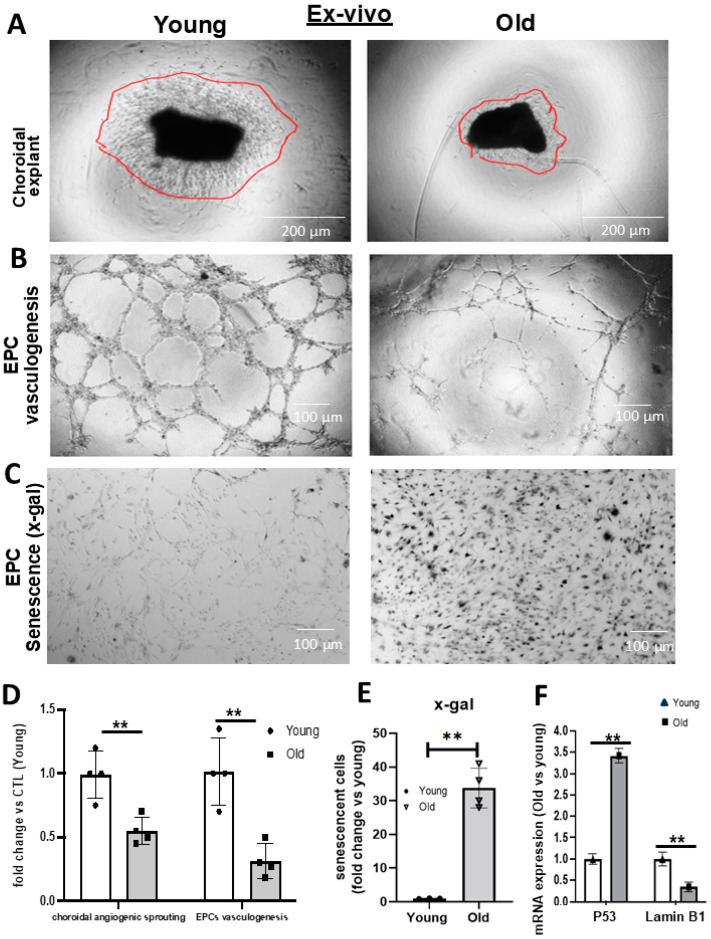
Comparison ex vivo of choroidal angiogenic sprouting, EPC vasculogenic capacity and senescence level in young vs. old rats. (**A**,**B**,**D**) Representative images and quantitative analysis of the angiogenic capacity of isolated choroidal explants (**A**,**D**) at day 5 and (**B**) EPCs at 6 h, derived from young vs. old rats, as assessed by the Matrigel assay. (**C**,**E**) EPC senescence level assessed by x-gal assay. (**F**) qRT-PCR analysis of P53 and Lamin B1 expression. Data were mean ± SEM. ** *p* < 0.01 vs. young rats. *n* = 5.

**Figure 3:**
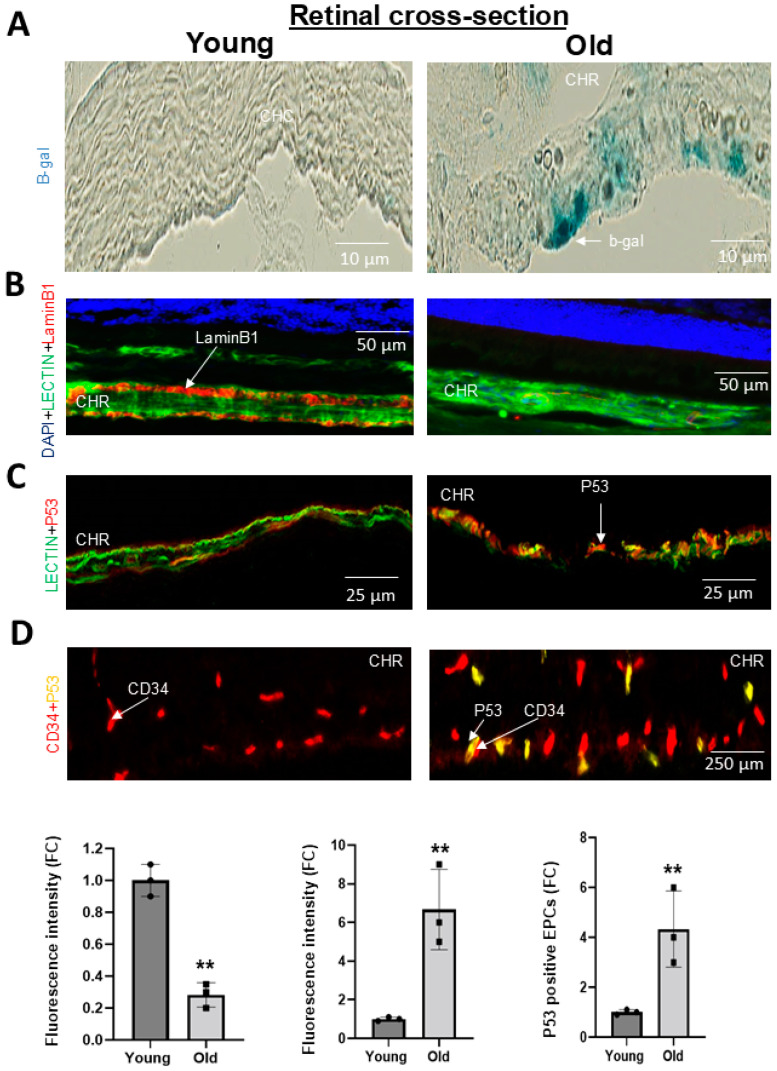
Senescence level in the choroid and in choroidal EPCs in young compared to old rats. (**A**,**B**) Representative images and quantification analysis of β-gal expression and LaminB1 in the choroidal vessels of young vs. old rats. (**C**) Representative image of p53 expression localized on choroidal vessels. (**D**) Representative image of p53 expression in EPCs co-localized in choroidal vessels. The white vertical bar in panel A identifies the thickness of the choroid (CHR). The image for the (**B**) panel in old animals is magnified 10-fold compared to images for young animals to compare the expression of p53 per same vessel area. The fluorescence intensity was measured for the stained markers (P53 and laminB1) per total choroid area using ImageJ. The histogram insets refer to expression profiles normalized to the choroids of young rats. Data were mean ± SEM. ** *p* < 0.01 vs. young rats. *n* = 5.

In a model of ROP involving oxidative stress and inflammation akin to conditions in aging, we had found that rats subjected to O_2_-induced retinopathy (OIR) presented a reduced number of CD34+ EPCs in their retinal vessels [14]. In the present study, we found, in older rats, a significant ~30% decrease in choroidal thickness beyond 0.6 mm of the optic nerve to 2.4 mm of the optic nerve (Figure 1A,C). A concomitant reduction in the thickness of the choroidal circulation-dependent outer nuclear layer (ONL) over >3.6 mm from the optic nerve was also observed in old rats (Figure 1A,C). In an attempt to understand the inability to restore the choroid, we studied the abundance of EPCs (using CD34 as well as CD133 as markers (Supplementary Figure S1)). We detected significantly fewer CD34+ and CD133+ cells adjusted to vessel area in the choroid of older rats (Figure 1B,D and Supplementary Figure S1). We next analyzed the vascular sprouting ability of a young vs. old choroid, and the vasculogenic capabilities of EPCs. We found that the choroid of old rats displayed negligible sprouting ex vivo in Matrigel (Figure 2A,D); likewise, bone marrow-derived EPCs from older subjects exhibited limited vasculogenesis (Figure 2B,D), consistent with a lower mRNA expression of EPC markers (CXCR4, CD34, CD117, CD133, and KLF-2) (Supplementary Figure S2A). This deficient ability of the choroid and EPCs from old rats to proliferate also inferred senescence. Concordantly, x-gal staining (a marker of senescence) was abundant on cultured EPCs from old rats (Figure 2C,E), and was associated with increased levels of p53 and the downregulation of Lamin B1 (Figure 2F); also, prominent tumor suppressor genes (p53, p16) involved in senescence were upregulated in older choroids (Supplementary Figure S2B). Additionally, the in situ staining of older choroids for senescence markers (increased β-gal and p53, and decreased Lamin B1) was confirmatory including in EPCs (Figure 3; see the phase contrast histological details in Supplementary Figure S3). Collectively, these results suggest that the choroidal involution observed in older rats seems to be mediated by a curtailed number of EPCs associated with a senescence phenotype.

### Age-Related Alterations in EPC Cellular Pathways

**Figure 4:**
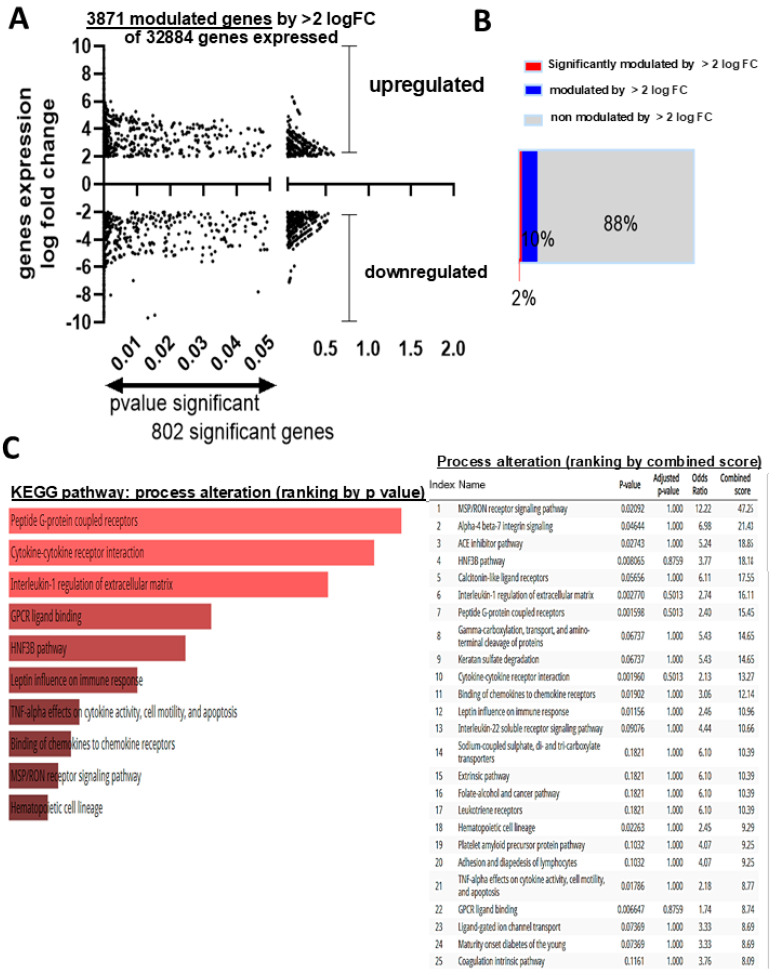
Global NGS analysis showing modulated genes and the associated altered pathways in EPCs derived from old rats. (**A**,**B**) Global evaluation of significant (*p* < 0.05) modulated gene (mRNA) expression levels (arbitrary cut-off levels set at a 2-log-fold change) in EPCs derived from old rats compared to young rats (**A**) and graphical representation in terms of the percentage of the distribution of the modulated genes in the whole genome (**B**). (**C**) Bioinformatic analysis identifying pathway enrichment (ranking by *p* value probability) by the modulation of the 802 mRNAs identified; the table on right side ranks pathways based on a combined score (of z-value deviation × the log of the *p* value score). The NGS data set represents the mRNA expression level in EPCs extracted in *n* = 3 rats per group.

To understand the underlying molecular mechanisms altered by aging which could be involved in the dysfunction of EPCs, we performed next-generation sequencing (NGS) in old and young EPCs. The NGS analysis identified 3871 gene transcripts (~2% of the total genes) modulated 2-logFC (log fold change) in old EPCs (Figure 4A,B); of these genes, 802 were significantly affected (*p* < 0.05). Of the top 50 genes modulated in old EPCs, most were transcription factors involved in various physiological or pathological processes (Supplementary Figure S4). Bioinformatic analysis detected age-related conditions; notably, inflammation (cytokine/receptor interaction and IL-1 and TNF signaling), GPCR signaling modulation, and hematopoietic cell lineage phenotype as critical pathways based on *p* value ranking (Figure 4C). Among the GPCRs, we identified five gene transcripts that were upregulated (GPCR-101, -152, -139, -157, -179) and two that were downregulated (GPCR-137b, -19) by ≥2 log FC (Supplementary Figure S5A); these receptors participate in various immune, cell differentiation, stress response coordination, and neuronal functions [22]. Whereas, among the inflammatory factors, numerous (specifically 16) pro-inflammatory cytokine genes were upregulated including IL-1α (Supplementary Figure S5B). Together these functions can participate in triggering or sustaining senescence [23,24].

**Figure 5:**
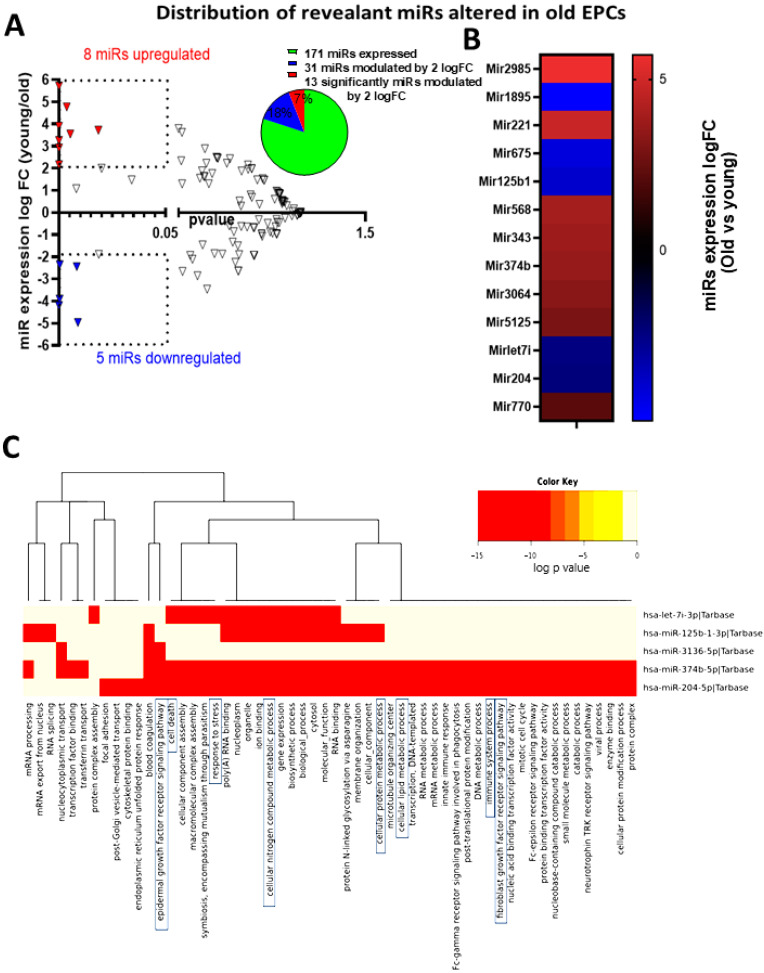
NGS analysis of the effect of aging on the expression level of miRNAs in EPCs and the associated pathway altered. (**A**) Global evaluation of significant (*p* < 0.05) modulated miRNAs (arbitrary cut-off levels set at a 2-log-fold change) in EPCs derived from old rats compared to young rats and a graphical representation in percentage terms of the miRNA distribution. (**B**) Heat map showing the identity and the logFC of the 13 miRNAs identified as significantly modulated. (**C**) KEGG analysis to identify predicted pathways altered (ranked by the *p* value of probability) by the simultaneous modulation of the five modulated miRNAs identified to be also expressed in humans. Blue box represent the pathway that potentially affect EPCs function. The NGS data set represents miRNA expression levels in EPCs extracted in *n* = 3 rats per group.

Additionally, the miRNA profile likely implicated in regulating gene expression was also performed by NGS on EPCs from young and old animals. Of the 171 miRNAs expressed in young EPCs, we focused on those most significantly modulated by aging. Using an arbitrary modulation expression cut-off level of a 2-log-fold change, we compared expression in young and old subjects, and found 13 age-modulated miRNAs, of which eight were upregulated and five downregulated, as depicted in Figure 5A and specifically identified in Figure 5B; these corresponded to ~7% of the expressed miRNAs. Interestingly, 5 of the 13 miRNAs were found to be conserved between species, including humans, and were also predicted to be associated with the modulation of major signaling pathways as per bioinformatic algorithms. Cell pathway-predicted KEGG analysis of the five interspecies-conserved miRNAs (specifically miR-125b1, miR-3136, miR-374, miR-204, and let71) suggests 46 pathways/processes to be affected by these miRs. As expected, several of the miR-modulated pathways are consistent with age-related choroidal involution, such as inflammation, cell endoplasmic reticulum stress response, growth factor, and catabolic modulation, which are major participants in cell senescence (Figure 5C). The modulation profiles of the predicted relevant targets of the three upregulated miRNAs conserved between species are presented in Supplementary Table S2.

## 4. Discussion

Aging is a physiological process associated with a plethora of diseases such as cancer; atherosclerotic-related events, including stroke and infarct; neurodegenerative pathologies; and, relevant herein, ischemic retinopathies such as AMD and diabetic retinopathies [25,26]. The choroid is a critical vascular tissue of the eye as it displays the highest blood flow in the body and is the exclusive supply of O_2_ and nutrients to photoreceptors. The ROP and AMD ocular pathologies, respectively, affect the extremes of life, and are characterized by an uncontrolled gradual degeneration of the choroidal vascular network resulting in photoreceptor dysfunction. Despite outer retinal hypoxia, in contrast to the retina, the choroid is limited in restoring its vascular network [18]. The gradual decline in choroidal tissue integrity has been found to involve the accumulation of replicative and non-replicative senescence, respectively, of the choroid and the retinal pigment epithelium [18]. Over the past few years, the concept of post-hypoxic/ischemic revascularization has evolved to involve both the mature ECs present locally in ischemic tissues and bone-marrow-derived CD34+ EPCs (also named pro-angiogenic cells). EPCs can act in a paracrine manner by secreting vascular growth factors or integrate into new vessels (vasculogenesis). Patients presenting cardiovascular risk factors along with aging display fewer circulating EPCs, which furthermore exhibit impaired vasculogenic activities [27,28]. Interestingly, similar profiles are observed in models of OIR in young animals subjected to comparable oxidative/inflammatory stress [29]. We proceeded to investigate the impact of aging on the density and senescent state of EPCs in the choroid of old vs. young subjects, and explored the age-related modulation of mRNAs and miRNAs using unbiased NGS.

We postulated that a decreased number and/or dysfunction of EPCs could be involved in age-related choroidal involution. This hypothesis was supported by the fact that the senescence-coupled aging of the choroid is associated with inflammation and oxidative stress, two factors known to impair EPC function [29]. Concordantly, we observed not only that the choroids of aged rats (~18 months) were relatively involuted compared to those of young animals (~6 months), but that they likely resulted in the thinning of the outer photoreceptor layer. Thinner aging choroids also displayed fewer EPCs. These findings were consistent with the reduced presence of EPCs in the vasculature of the ischemic muscles in aging animals [14,30], as well as in the circulation of older human patients, presenting risk factors for cardiovascular diseases [27,30]. We also found that older choroids and EPCs display poor angiogenic ability.

A major feature in the present study is that impairment in choroidal vascular proliferation was at least in part mediated by endothelial and endothelial progenitor cell senescence, as attested by deficient cell replication and established markers of senescence. Our results show high levels of senescence in the aged choroidal vessels and in EPCs that are localized in the choroid. Senescence is an adaptative cellular response in mitotic and post-mitotic cells that is activated by several factors such as the tumor suppressor proteins and the inflammatory secretome [31]. The accumulation of senescent cells leads to altered metabolism, increased generation of ROS, and polarization towards a pro-inflammatory secretary phenotype, the key elements involved in vascular degeneration. In this context the major cell fate-determinant tumor suppressor protein p53 is upregulated in EPCs; the silencing of p53 in EPCs before transplantation has been shown to improve the formation of collateral vessels in a model of diabetic vaso-occlusive disease [32] and restore choroidal thickness in OIR [18]. Concomitant to increased expression of p53, the age-dependent loss of Lamin B1, an established marker that wanes during cellular senescence, is associated with the impaired function of adult neural stem progenitor cells and reduced physiological neurogenesis in the hippocampus [33], in line with the choroidal involution observed herein.

NGS made it possible to further understand the modulation of genes (mRNAs) and the potential contribution of miRNAs to the age-related impairment of EPCs [34]. We used the bioinformatic KEGG pathway analysis of modulated genes to determine the potentially implicated expression of major processes, notably GPCR signaling and inflammation [35]. The precise role of GPCRs in EPC functional activity remains to be elucidated, but this large receptor family has been involved in transmitting signals between the extra- and intra-cellular compartments to elicit a large scale of biological responses including physiological and pathological angiogenesis/vasculogenesis [36]. For instance, in diabetic retinopathies, the activation of GPR91, a succinate-specific receptor, initiates the signal transduction that leads to the upregulation of pro-inflammatory and pro-angiogenic cytokines [37]. In another study, GPR91 was shown to promote the expression of VEGF through ERK signaling, which in turn leads to the breakdown of the blood–retinal barriers [38]. In the context of aging, it has also been reported that the expression of cerebrovascular endothelial GPCRs is altered, and contributes to endothelial dysfunction [39]. Other studies have highlighted the importance of GPCRs in regulating the function of stem cells during embryonic development [40], cell survival [41], and importantly cell migration and homing functions through the regulation of SDF-1/CXCR4 signaling [42,43], a key pathway involved in the migration and homing of EPCs to ischemic tissues [44]. Hence, based on the biological properties of diverse GPCRs, GPCR pathway alterations can contribute to the impairment of EPCs.

NGS also detected an age-related upregulation in the expression of inflammatory mediators, particularly IL-1. As this specifically pertains to the choroid, we have previously reported that inflammation is a major participant in OIR-associated choroidal involution and senescence [18]. Our present results show that several important members of the interleukin family, including IL-1α, are upregulated in older EPCs. IL-1 is not only linked to endothelial dysfunction, but its pathogenic role is well known in ischemic retinopathies like ROP [45] and AMD [46]. The inflammatory process is strongly associated with dysfunction of mature endothelium and EPCs, such that inflammation and senescence are intertwined; increased expression of IL-1 can drive cells into a state of pseudo-senescence [47]; and inhibition of IL-1 can restore choroidal integrity (in a model of OIR) [5]. The signaling of IL-1-generating inflammasome can also induce and maintain senescence [48] through direct effects as well as by promoting the secretion of other major inflammatory factors [49], namely IL-6 and IL-8 [50]. Likewise, inflammation-ridden senescence-associated secretory phenotype (SASP) can trigger senescence in nearby cells [16]. In addition, modulated GPCRs can affect the inflammatory processes. Such is the case for GPR101 in macrophages which affects host immune response [51]; meanwhile, GPR137 can inhibit cellular proliferation and promote neuronal differentiation in tumor cells [52].

To further understand the changes linked to aging of EPCs, we performed expression profiling of miRNAs, which are endogenous small non-coding RNAs (20–25 nucleotides) that regulate translation of mediators for a wide range of physiological and pathological processes such as angiogenesis, inflammation, and senescence, including in EPCs [10,14,19,53]. For instance, of the 13 age-modulated miRNAs the upregulated miR-221 has been reported to negatively regulate EC function and angiogenesis [54]. miR-221 has also been shown to suppress HIF-1, limit angiogenesis during heart failure [55], and inhibit the proliferation of EPCs. Interestingly, another study reported that miR-221 inhibits the transition of EPCs to MSCs via the PTEN/Fox02a pathway [56]. Our results also demonstrate that miR-675, a miRNA strongly associated with the regulation of senescence [57], is downregulated in older EPCs. In contrast, the downregulation of let7i observed in our study could also alter the angiogenic function of EPCs by suppressing their migratory activity; let7i enhances cell migration and proliferation by targeting HABP4 in renal carcinoma cells [58]. Finally, we identified using bioinformatic prediction analysis that the combined actions of different miRNAs can affect various pathways involved in angiogenesis, immune response, and cell death.

In conclusion, we hereby report that age-related choroidal involution is associated with reduced numbers of CD34+/CD133+ EPCs localized in choroidal vessels, and that the EPCs of older rats exhibit a senescent-like phenotype; this association may or may not establish a causal relationship. Altogether, our NGS results suggest that the mechanisms associated with the dysfunction of aged EPCs are diverse, including the activation of the inflammasome and the dysregulation of cell-proliferative and well-being-related GPCRs, and affected by altered expression of related miRNAs. We propose that biological modification of native EPCs (such as with senolytic agents) could provide a novel strategy to preserve the vascular integrity of the aging choroid and prevent development of maculopathies in older subjects.

## Data Availability

Data are available on request to the correspondent author at micheldesjarlais@gmail.com.

## Supplementary Materials

The following supporting information can be downloaded at: https://www.mdpi.com/article/10.3390/biomedicines12122669/s1, Figure S1. Representative immunohistochemical image of choroid of young and old rats, showing CD133 expression (red); co-localization with lectin (green) is full as reflected in yellow stain. Vertical scale bar for old rat choroid is magnified 10-fold to make it possible to compare expression over the same area. Figure S2. Heat map of the expression level of EPC markers and senescence-associated genes in the choroid of young vs. old rats. (A,B) qRT-PCR analyses of the expression level of EPCs markers (A) and senescence-associated genes (B) in the choroidal tissues of old vs. young rats. Data are mean ± SEM. * *p* < 0.05 or ** *p* < 0.01 vs. young rats. *n* = 3. Figure S3. Phase contrast representative microscopic images of outer and sub-retina stained for β-gal. Figure S4. Individual NGS analysis showing the top modulated genes. An overview of the top 50 modulated genes in old EPCs. The NGS data set represents mRNA expression levels in EPCs extracted from *n* = 3 rats per group and expressed as a log-fold change (logFC) of old vs. young rats. Figure S5. Individual NGS analysis for GPCR and interleukin families. (A) modulated genes in the GPCR family and (B) interleukin. The NGS data set represents mRNA expression levels in EPCs extracted from *n* = 3 rats per group and expressed as a log-fold change (logFC) of old vs. young rats. Figure S6. Measurements of the thickness of the choroidal membrane and the distance between specific areas. Table S1. List of primer sequences used for qRT-PCR. Table S2. List and expression profile (using NGS data) of relevant predictive targets (using target scan V8) of three upregulated miRs in choroids of old rats.
