# Supplementary material for: Age-Related Choroidal Involution Is Associated with the Senescence of Endothelial Progenitor Cells in the Choroid
Source: Biomedicines. 2024 Nov 22;12(12):2669. doi: 10.3390/biomedicines12122669 (PMC11726740; doi:10.3390/biomedicines12122669)
Supplement: Supplementary file 1 [file biomedicines-12-02669-s001.zip › biomedicines-3288049-supplementary.pdf]

biomedicines

MDPI

Supplementary Materials:

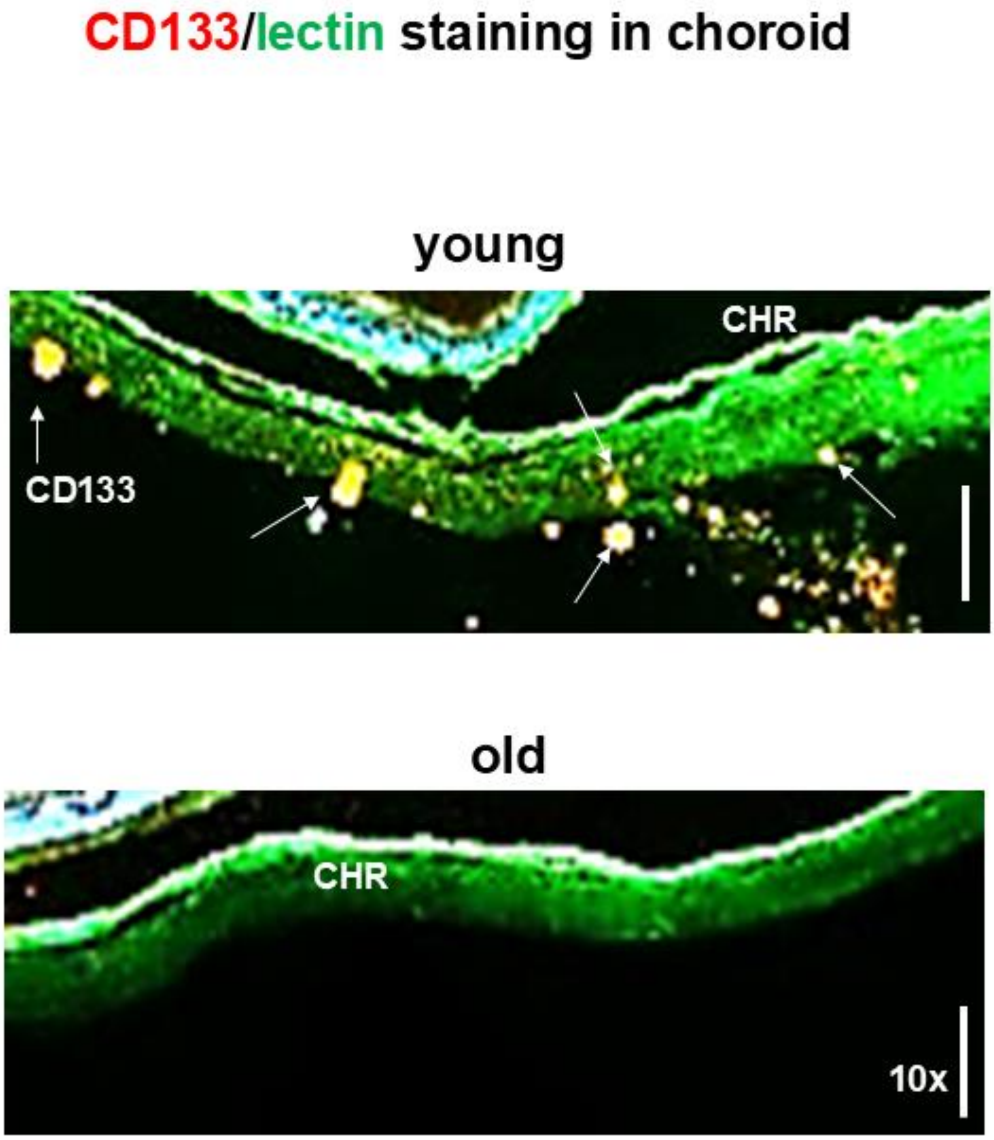

Supplemental Figure S1. Representative immune-histochemical image of choroid of young and old rats, showing CD133 expression (red); co-localization with lectin (green) is full as reflected in yellow stain. Vertical scale bar for old rat choroid is magnified 10-fold to allow to compare expression over the same area.

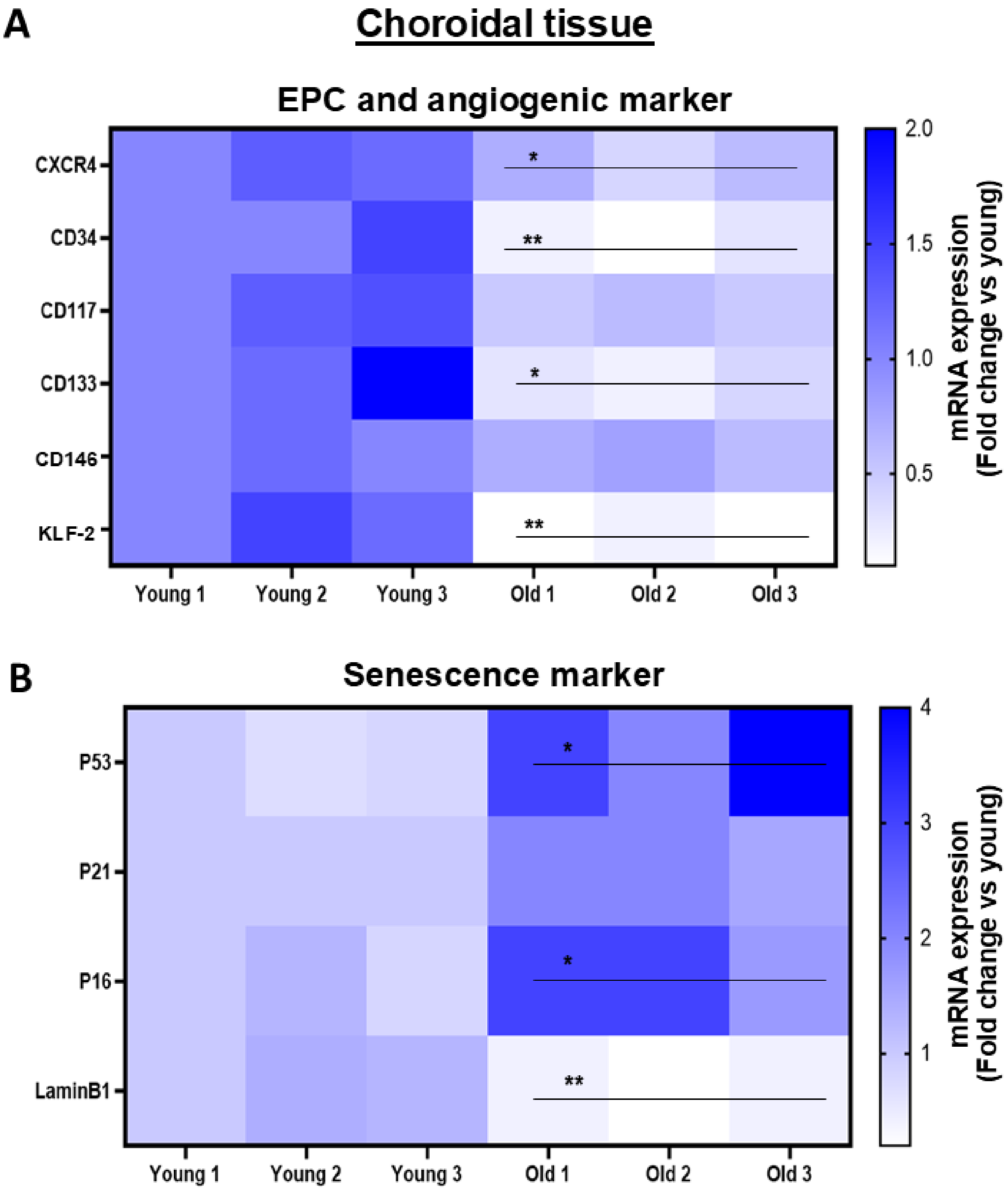

Supplemental Figure S2. Heat map of the expression level of EPC markers and senescence-associated genes in the choroid of young vs old rats. (**A**,**B**) qRT-PCR analyses of the expression level of EPCs markers (**A**) and senescence-associated genes (**B**) in choroidal tissues of old vs young rats. Data were mean ± SEM. * p<0.05 or ** p<0.01 vs young. N=3.

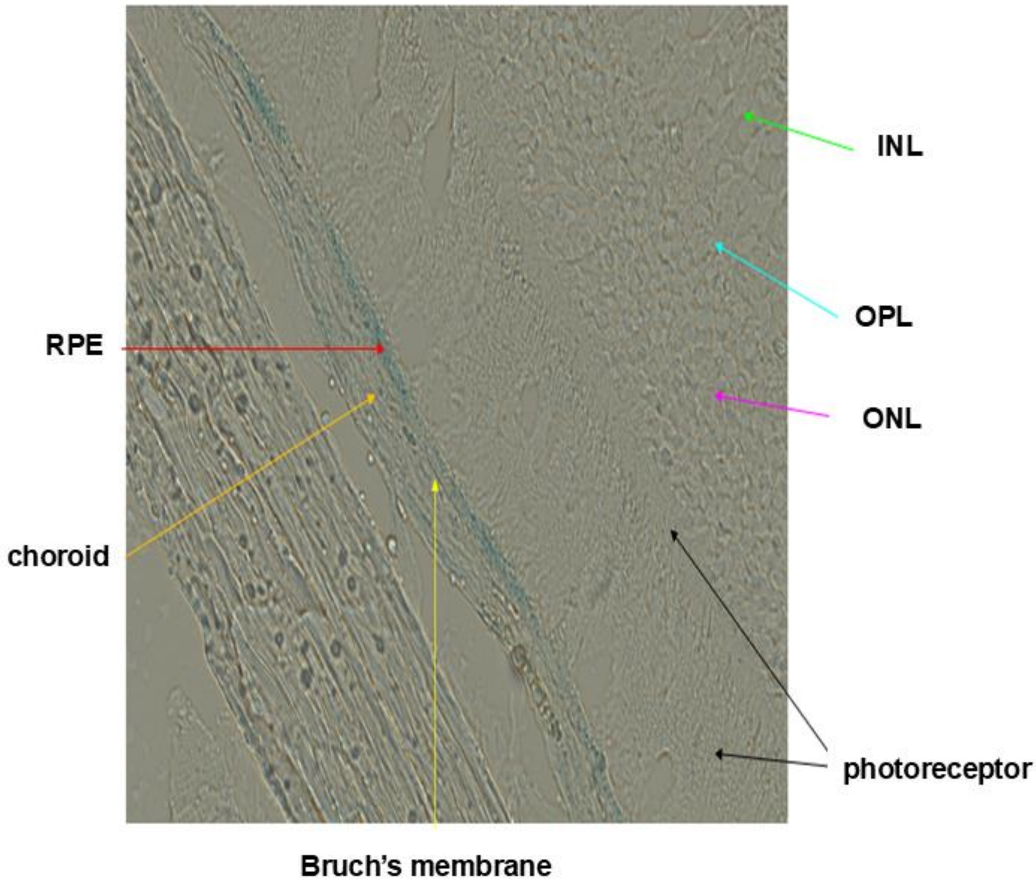

Supplemental Figure S3. Phase contrast representative microscopic images of outer and sub-retina stained for β-gal.

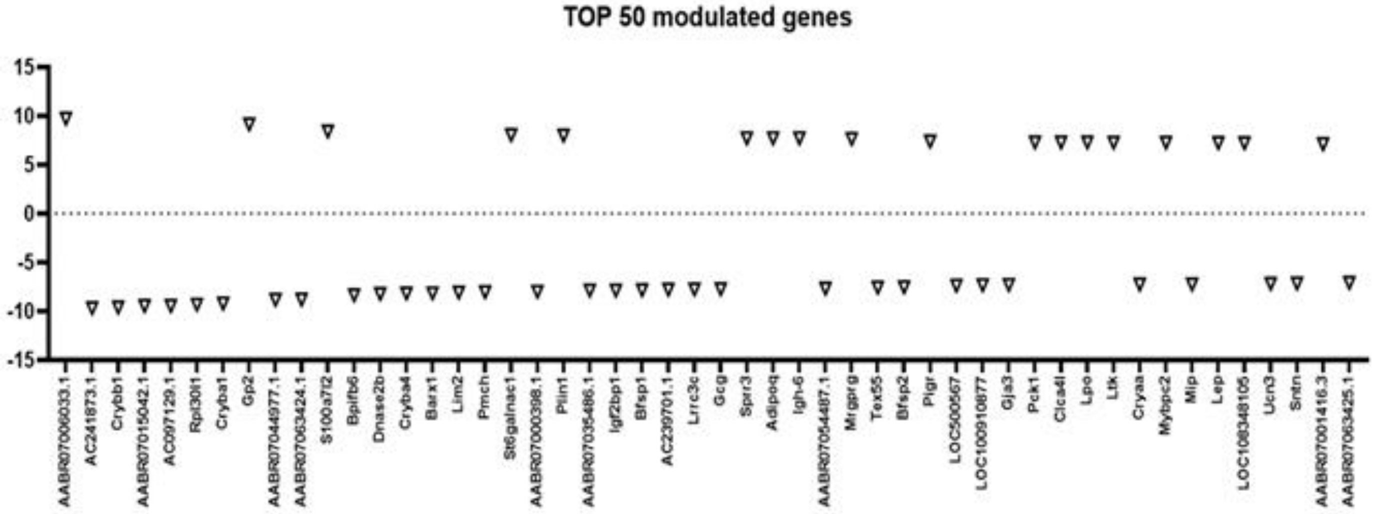

Supplemental Figure S4. Individual NGS analysis showing the top modulated genes. Overview of the top 50 modulated genes in old EPCs. The NGS data set represent mRNA expression level in EPCs extracted in N=3 rats per group and expressed as log-fold change (logFC) of old vs young rats.

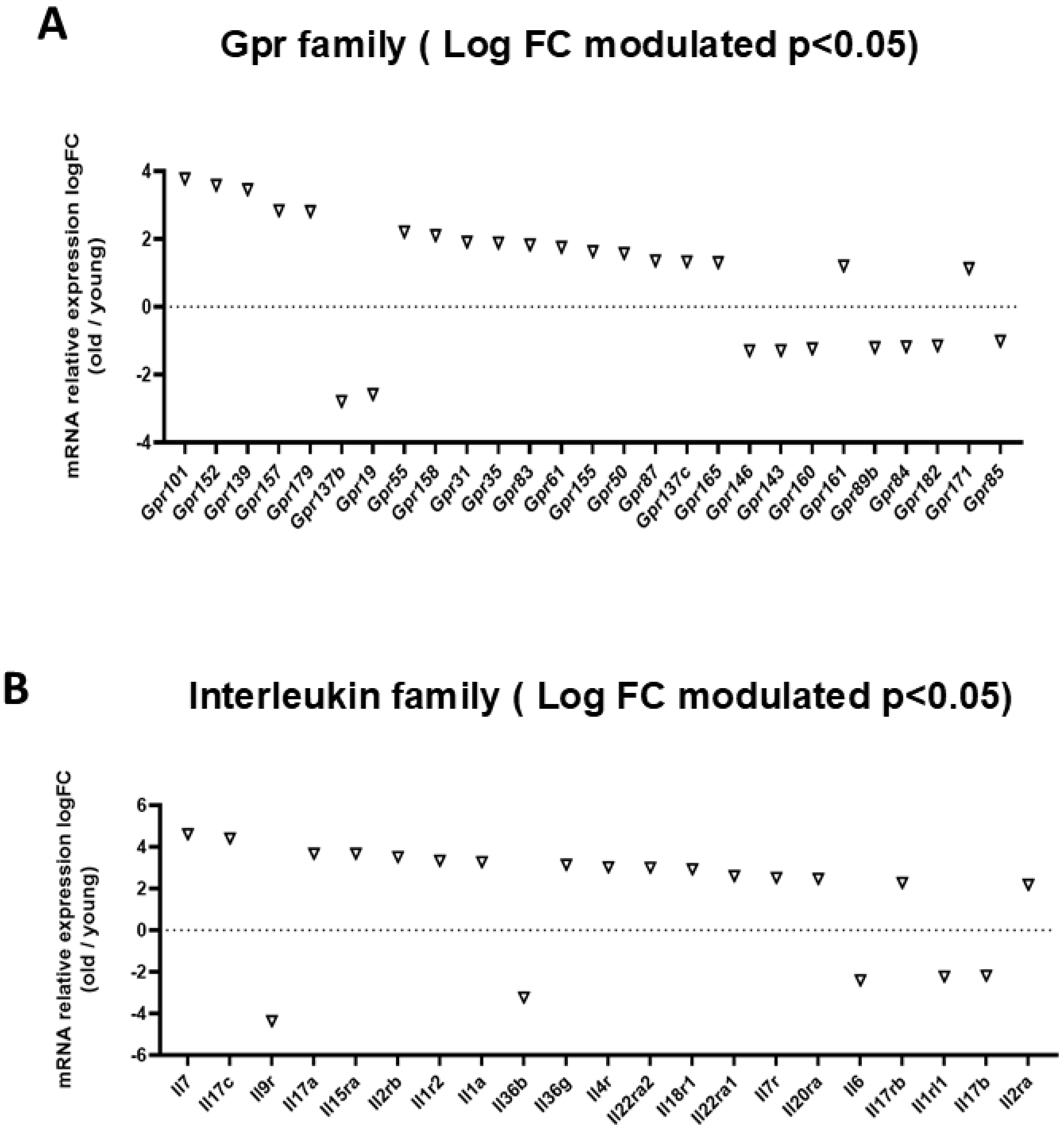

Supplemental Figure S5. Individual NGS analysis for GPCR and interleukin families. (**A**) modulated genes in the GPCR family and (**B**) Interleukin. The NGS data set represent mRNA expression level in EPCs extracted in N=3 rats per group and expressed as log-fold change (logFC) of old vs young rats.

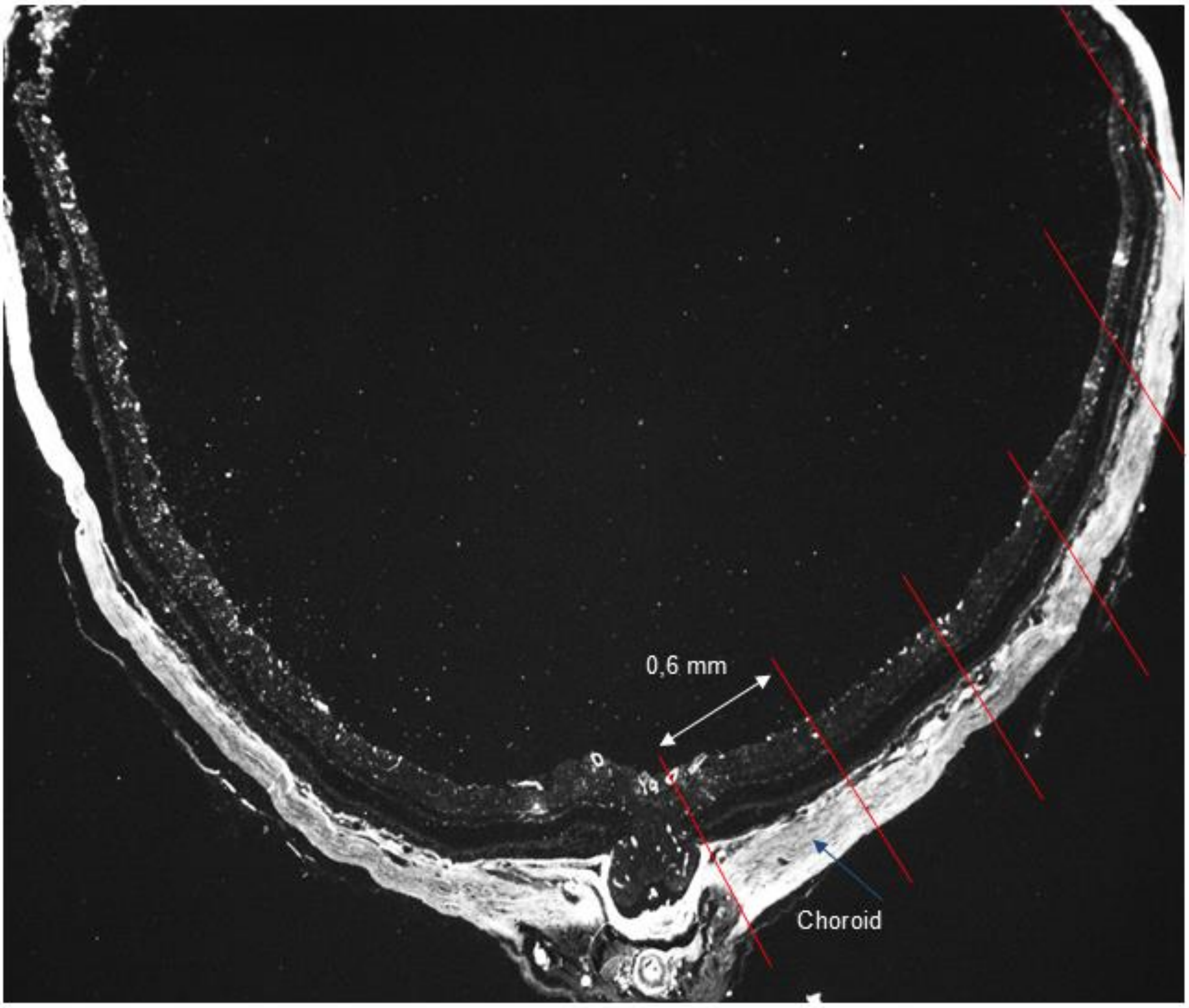

Figure S6. Measurements of the thickness of the choroidal membrane and the distance between specific areas.

Supplemental Table S1. List of primer sequences used for qRT-PCR.

	foward	reverse
CXCR4	GCCATGGCTGACTGGTACTT	GATGAAGGCCAGGATGAGAA
CD34	GTCACACTGCCTACTACTTC	TCCTCGGATTCCTGAACAT
CD117	ACATCGCCAGAGCCAACG	ATCCACTTTAATTTCGGGTCAA
CD133	GGACCCATTGGCATTCTC	CAGGACACAGCATAGAATAATC
KLF-2	TCGCACCTAAAGGCGCATC	TAGTGGCGGGTAAGCTCGTC
CD146	AGCTCCGCGTCTACAAAGC	CTACACAGGTAGCGACCTCC
P53	GTTCCGAGAGCTGAATGAGG	ACTTCAGGCTGGAGTGA
P21	GAGCAGTCCCGAGTTAAGG	TGGAACAGGTCGGACATCAC
P16	CCTCGTGCTGATGCTACTGA	TGGAACAGGTCGGACATCAC
LaminB1	TCTTCTGCCTCCAGTGTCACAG	CATGATGCTGCAGTTCTGGGAG
β-Actin	GTGGGCCGCACAAGGCACCAA	CTCTTTGATGTCACGCACGA
GAPDH	AGCCACATCGCTCAGACACC	GCGCCCAATACGACCAAA

Supplemental Table S2. List and expression profile (using NGS data) of relevant predictive targets (using target scan V8) of 3 upregulated miRs in choroid of old rats.

miR-221 (target)

Gene ID	Gene name	Process involved	Log Fold change (old/young)
CDKN1b	cyclin-dependent kinase inhibitor 1B	proliferation	0.17
BMF	Bcl2 modifying factor	apoptosis	2.29
**KLF7**	**Kruppel-like factor 7**	**EPC function**	**-0,84**
THBS1	thrombospondin 1	angiogenesis	1,91
FGF14	fibroblast growth factor 14	angiogenesis	0,43

miR-374b (target)

Gene ID	Gene name	Process involved	Log Fold change (old/young)
**CCL2**	**chemokine (C-C motif) ligand 2**	**migration**	**-3,44**
CCL8	chemokine (C-C motif) ligand 8	migration	na
**VWC2**	**von Willebrand factor C domain containing 2**	**angiogenesis**	**-0,62**
IL10	interleukin 10	inflammation	0,83
**MMP14**	**matrix metallopeptidase 14 (membrane-inserted)**	**angiogenesis**	**-0,69**

miR-770 (target)

Gene ID	Gene name	Process involved	Log Fold change (old/young)
SUCNR1	succinate receptor 1	metabolism	3,08
RRAGB	Ras-related GTP binding B	migration	na
**CDKL4**	**cyclin-dependent kinase-like 4**	**proliferation**	**-0,25**
GPR50	G protein-coupled receptor 50	angiogenesis	1,57
CDKAL1	CDK5 regulatory subunit associated protein 1-like 1	proliferation	na

Targetscan_V8
